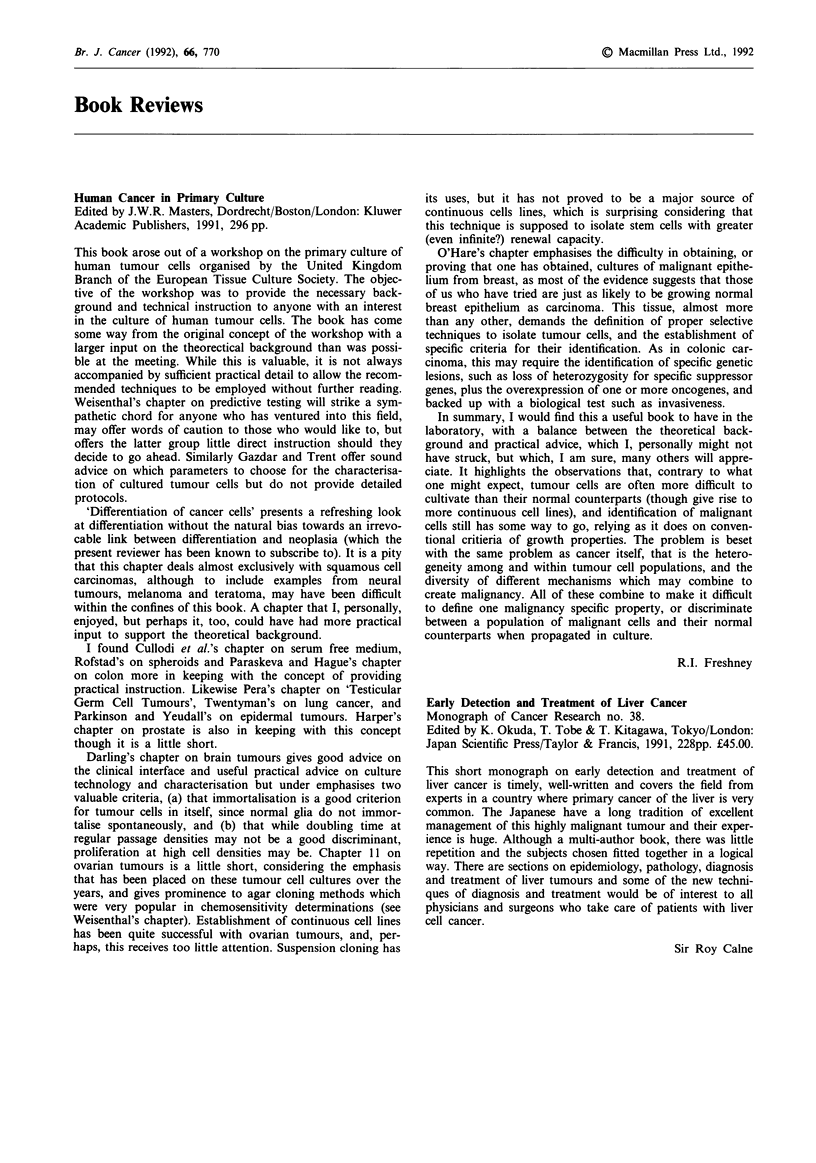# Early Detection and Treatment of Liver Cancer

**Published:** 1992-10

**Authors:** Roy Calne


**Early Detection and Treatment of Liver Cancer**
Monograph of Cancer Research no. 38.
Edited by K. Okuda, T. Tobe & T. Kitagawa, Tokyo/London: Japan Scientific Press/Taylor & Francis, 1991, 228pp. £45.00.

This short monograph on early detection and treatment of liver cancer is timely, well-written and covers the field from experts in a country where primary cancer of the liver is very common. The Japanese have a long tradition of excellent management of this highly malignant tumour and their experience is huge. Although a multi-author book, there was little repetition and the subjects chosen fitted together in a logical way. There are sections on epidemiology, pathology, diagnosis and treatment of liver tumours and some of the new techniques of diagnosis and treatment would be of interest to all physicians and surgeons who take care of patients with liver cell cancer.

Sir Roy Calne